# Crystal structures of (*S*)-(−)-1-(4-chloro­phen­yl)-*N*-[(pyridin-2-yl)methyl­idene]ethan-1-amine and its *cis*-di­chlorido­{(*S*)-(−)-1-(4-chloro­phen­yl)-*N*-[(pyridin-2-yl)methyl­idene]ethan-1-amine}palladium(II) complex

**DOI:** 10.1107/S2056989025011430

**Published:** 2026-01-08

**Authors:** Teresa Pacheco-Álvarez, Alejandro Yañez-Cabrera, C. Claudia P. Villamizar, Pankaj Sharma, Bertin Anzaldo, Angel Mendoza, Guadalupe Hernández Téllez

**Affiliations:** a Lab. Síntesis de Complejos, Fac. Cs. Quím. Benemérita Universidad, Autónoma de Puebla, Ciudad Universitaria, PO, Box, 72592 Puebla, Mexico; b Lab. Síntesis de Complejos, Fac. Cs. Quím., Benemérita Universidad Autónoma de Puebla, Ciudad Universitaria, PO Box 72592, Puebla, Mexico; c Instituto de Química Universidad Autónoma de México UNAM, Circuito Exterior Cd. Universitaria, PO Box 04510, Ciudad de México, Mexico; d Centro de Química, ICUAP, Benemérita Universidad Autónoma de Puebla, 72570 Puebla, Mexico; Vienna University of Technology, Austria

**Keywords:** crystal structure, Schiff base, palladium(II) complex

## Abstract

One of the title compounds, the chiral Schiff base (*S*)-(−)-1-(4-chloro­phen­yl)-*N*-[(pyridin-2-yl)methyl­idene]ethan-1-amine (C_14_H_13_ClN_2_) crystallizes in the monoclinic Sohncke space group *P*2_1_ with one mol­ecule in the asymmetric unit. The ligand is obtained by condensation of (*S*)-(−)-1-(4-chloro­phen­yl)ethan-1-amine with 2-pyridine­carboxaldehyde. Its palladium(II) chloride complex, *cis*-di­chlorido­{(*S*)-(−)-1-(4-chloro­phen­yl)-*N*-[(pyridin-2-yl)methyl­idene]ethan-1-amine-κ^2^*N*,*N*′}palladium(II), [PdCl_2_(C_14_H_13_ClN_2_)], crystallizes in the ortho­rhom­bic Sohncke space group *P*2_1_2_1_2_1_, with one mol­ecule in the asymmetric unit. The central Pd^II^ atom adopts a slightly distorted square-planar coordination environment, defined by two nitro­gen donors (pyridyl-N and imine-N) and two chlorido ligands in a *cis* arrangement.

## Abstract 2

Both title compounds crystallize in Sohnke space groups. The ligand crystallizes in *P*2_1_, and the complex in *P*2_1_2_1_2_1_, with the Pd^II^ atom in a slightly distorted square-planar environment.

## 1. Chemical context

Mol­ecules containing imine or azomethine C=N groups are widespread in chemical and biological systems. Schiff bases, formed readily by the condensation of aldehydes or ketones with primary amines, are synthetically accessible and structurally versatile (Anzaldo Olivares *et al.*, 2019 ▸; Hernández Téllez *et al.*, 2025 ▸). These features make them particularly valuable in coordination chemistry, enabling the rational design of various metal complexes (Dalia *et al.*, 2018 ▸). In *d*-block chemistry, Schiff-base ligands can stabilize metals in multiple oxidation states, while systematic modifications of the imine framework allows fine-tuning of electronic and steric properties (Takeda *et al.*, 2023 ▸).

Chelation governs many processes in organometallic and bioinorganic chemistry: steric factors strongly influence coordination, geometry and reactivity (Mandal & Pratihar, 2023 ▸; Fabbrizzi, 2020 ▸). In particular, Pd^II^ complexes are central in catalytic reactions; their kinetic reactivity often exceeds that of Pt^II^ analogues by 4-5 orders of magnitude (Bugarčić *et al.*, 2015 ▸).

Herein we report the synthesis and crystal structures of a chiral Schiff base, C_14_H_13_ClN_2_, and its corresponding PdCl_2_ complex, [PdCl_2_(C_14_H_13_ClN_2_)].
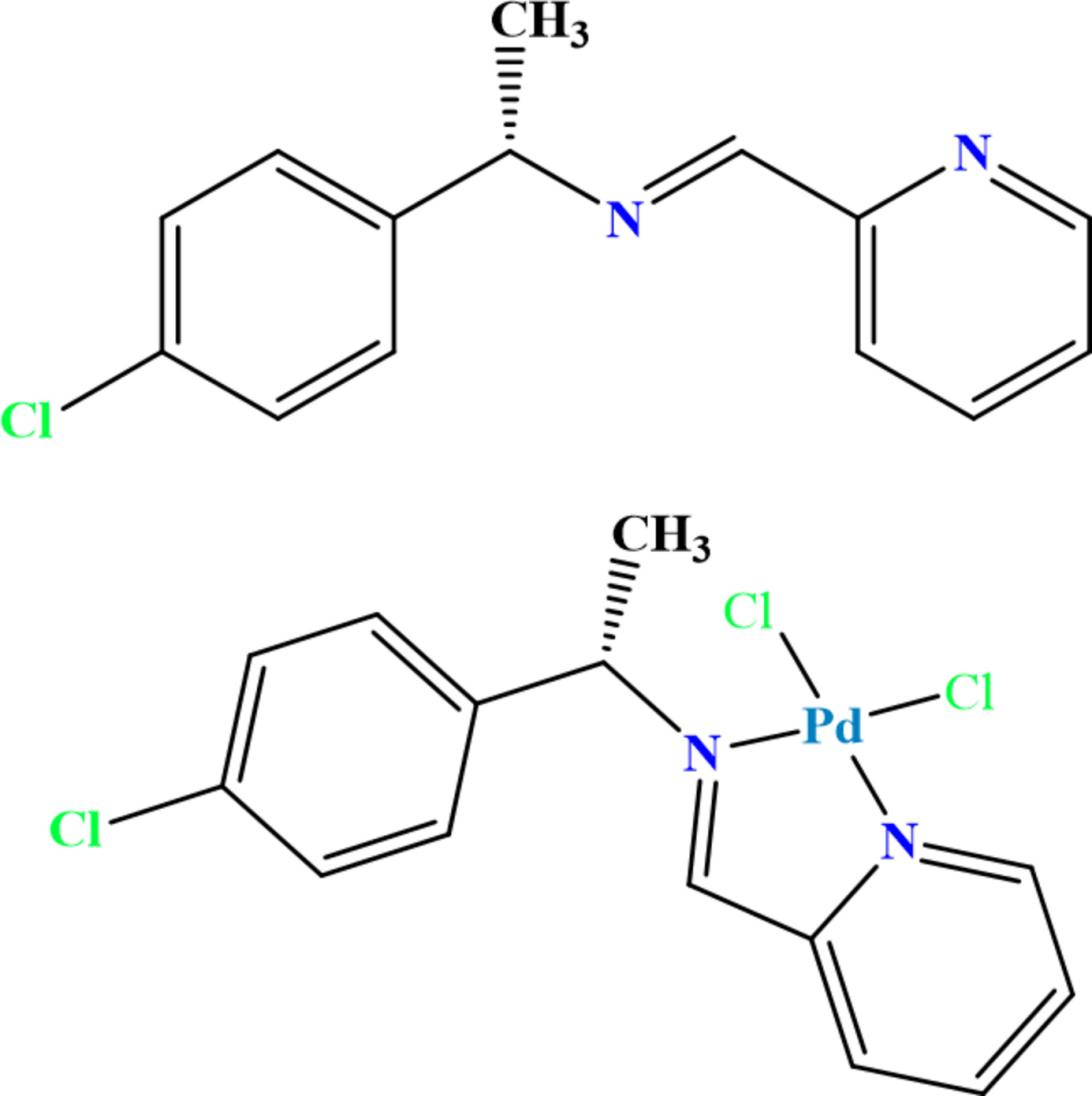


## 2. Structural commentary

**Figure 1:**
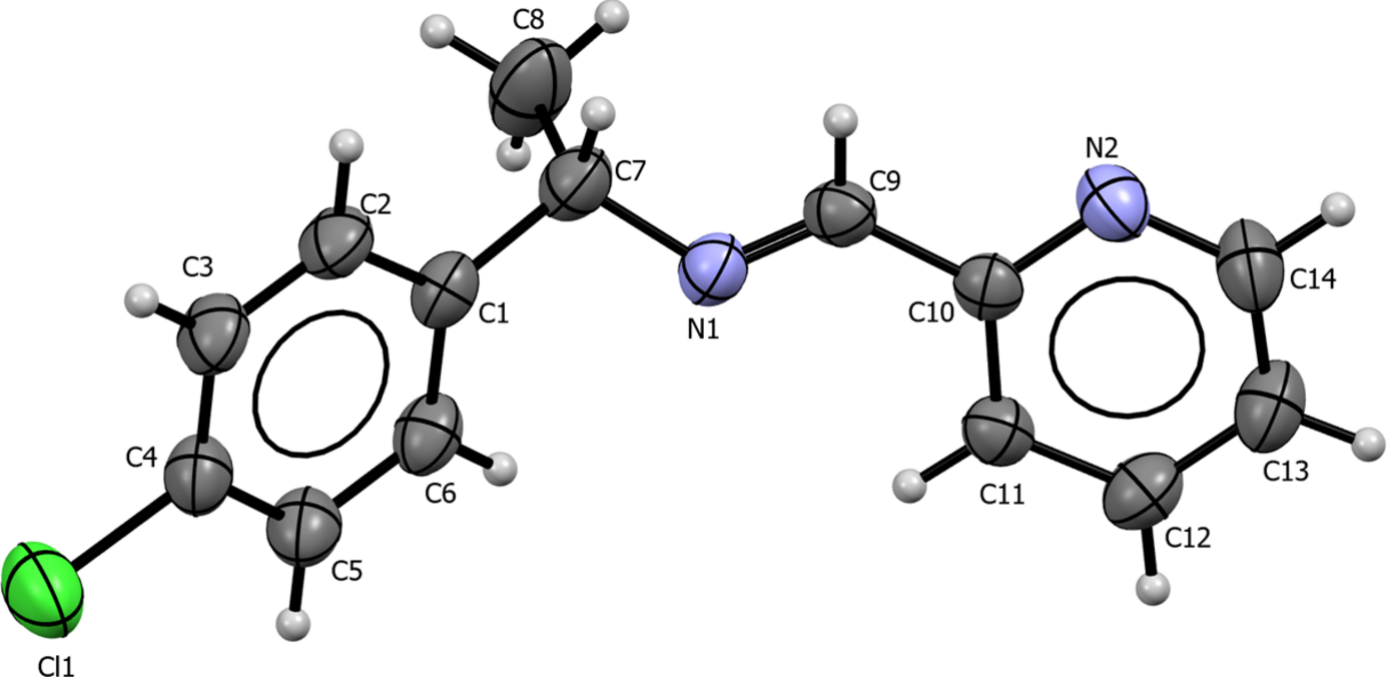
Mol­ecular structure of (*S*)-(−)-1-(4-chloro­phen­yl)-*N*-[(pyridin-2-yl)methyl­idene]ethan-1-amine. Displacement ellipsoids are drawn at the 30% probability level; H atoms are given as spheres of arbitrary radius.

The mol­ecular structure of the Schiff base ligand is shown in Fig. 1 ▸. The crystal belongs to the monoclinic Sohncke space group *P*2_1_ and the mol­ecule displays the *E* configuration about the C=N double bond and the *(S)* configuration at the stereogenic center. The free ligand exhibits an imine C9=N1 bond length of 1.253 (4) Å, within the expected range for >C=N bonds, and a C7—N1—C9 bond angle of 117.4 (3)°, consistent with *sp*^2^ hybridization at C9 and N1. The ligand adopts a conformation governed by steric repulsion: the two aromatic rings are not coplanar, the angle between the plane defined by ring N2–C14–C13–C12–C11–C10 and the plane defined by ring C6–C1–C2–C3–C4–C5 is 32.08 (16)°.

**Figure 2:**
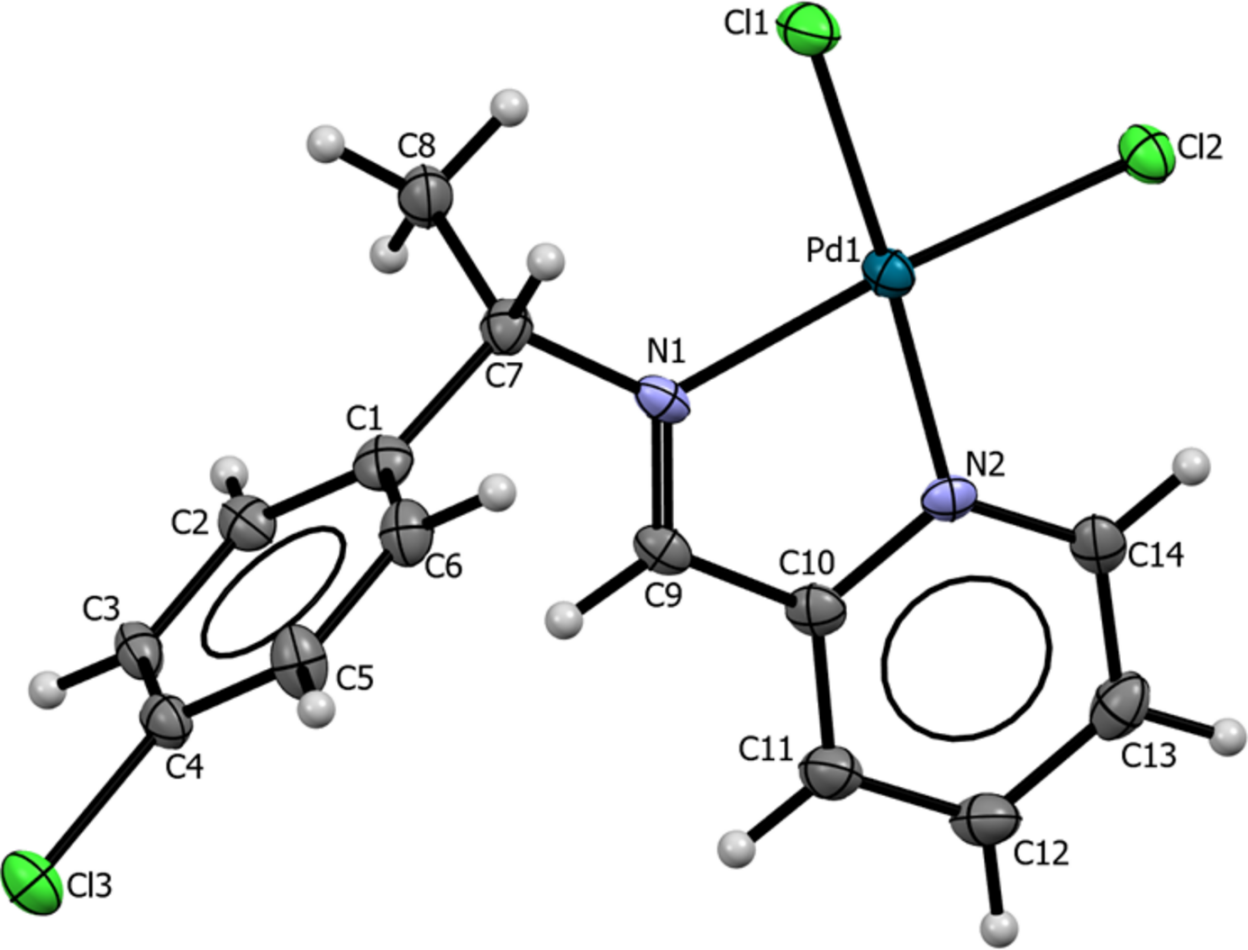
Mol­ecular structure of the di­chlorido­{(*S*)-(−)-1-(4-chloro­phen­yl)-*N*-[(pyri­din-2-yl)methyl­idene]ethan-1-amine}­palladium(II) complex. Displacement ellipsoids are drawn at the 30% probability level; H atoms are given as spheres of arbitrary radius.

The corresponding PdCl_2_ complex crystallizes in the ortho­rhom­bic Sohncke space group *P*2_1_2_1_2_1_, with one mol­ecule in the asymmetric unit. The imine ligand bonds to the Pd^II^ atom via two nitro­gen donors (imine N1 and pyridyl N2) within an N^N five-membered chelate ring. Together with the two chlorido ligands, a distorted square-planar coordination is realized (Fig. 2 ▸), with an N1—Pd—N2 bite angle of 80.8 (3)°. Observed bond lengths are Pd1—N1 = 2.045 (8) Å, Pd1—N2 = 2.036 (8) Å, Pd1—Cl1 = 2.291 (3) Å and Pd1—N2 = 2.282 (3) Å, values consistent with closely related Pd(II) complexes. The Cl1—Pd—Cl2 angle of 90.85 (9)° approximates the ideal 90° for ideal square-planar coordination. The imine bond in the complex, C9=N1 = 1.287 (10) Å, is slightly longer than in the free ligand and, together with a C7—N1—C9 angle of 122.0 (8)°, is consistent with *sp*^2^ hybridization. The Pd(II) atom lies 0.012 Å out of the mean N_2_Cl_2_ coordination plane. Coordination to the metal increases steric effects in the ligand backbone, reflected by the change in torsion angle C1—C7—N1—C9 from −142.4 (3)° in the free ligand to 24.3 (12)° in the complex. Weak intra­molecular C—H⋯Cl inter­actions stabilize the mol­ecular conformation (Table 1 ▸; entries 3–5)

## 3. Supra­molecular features

**Figure 3:**
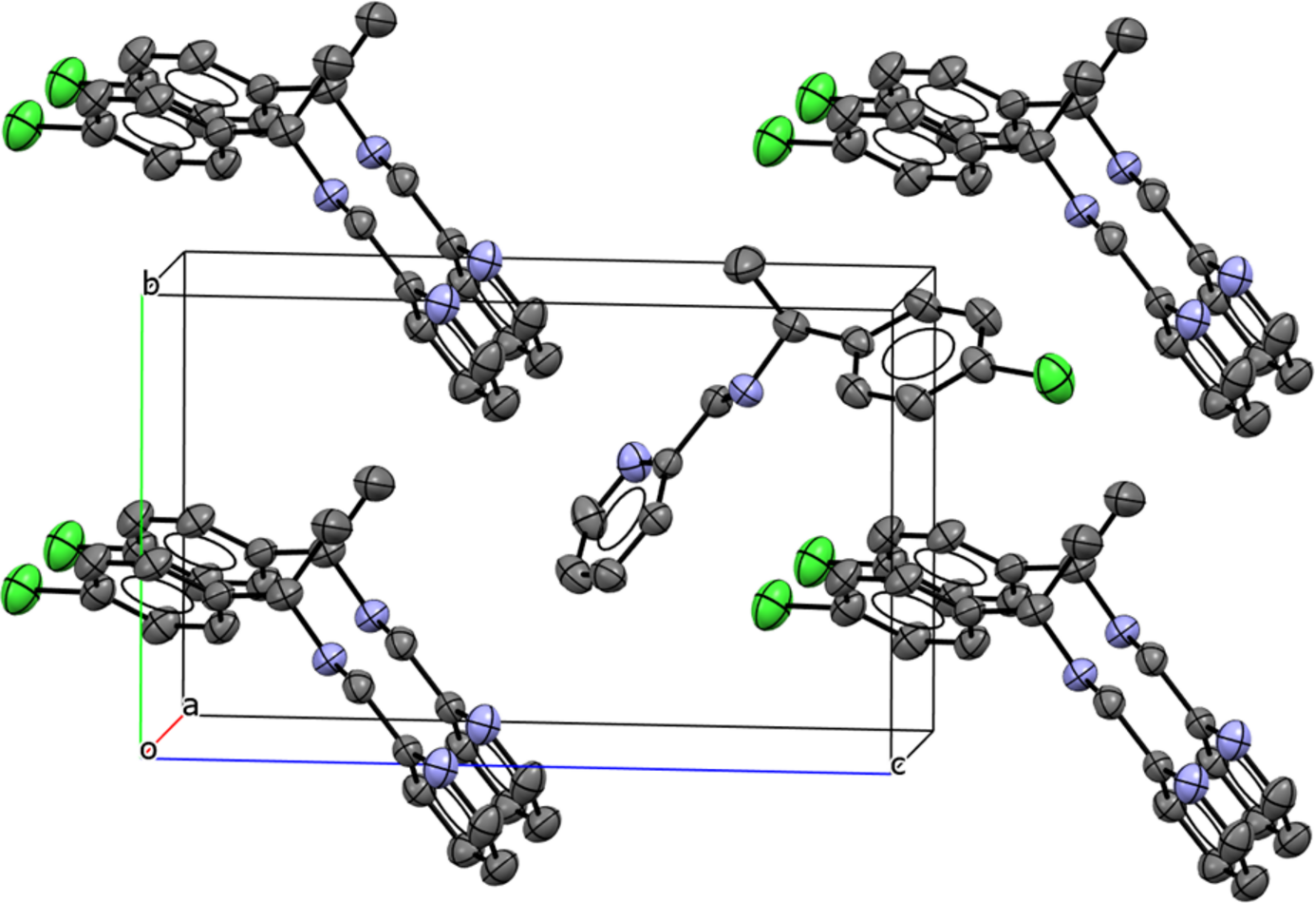
Crystal packing of the imine ligand. Displacement ellipsoids are as in Fig. 1 ▸; all H atoms have been omitted for clarity.

Packing analysis of the imine ligand reveals no significant hydrogen-bonding inter­actions, and π–π stacking is negligible: centroid-to-centroid separations exceed 4.50 Å, larger than the typical range of 3.30–3.80 Å for these inter­actions (Fig. 3 ▸).

**Figure 4:**
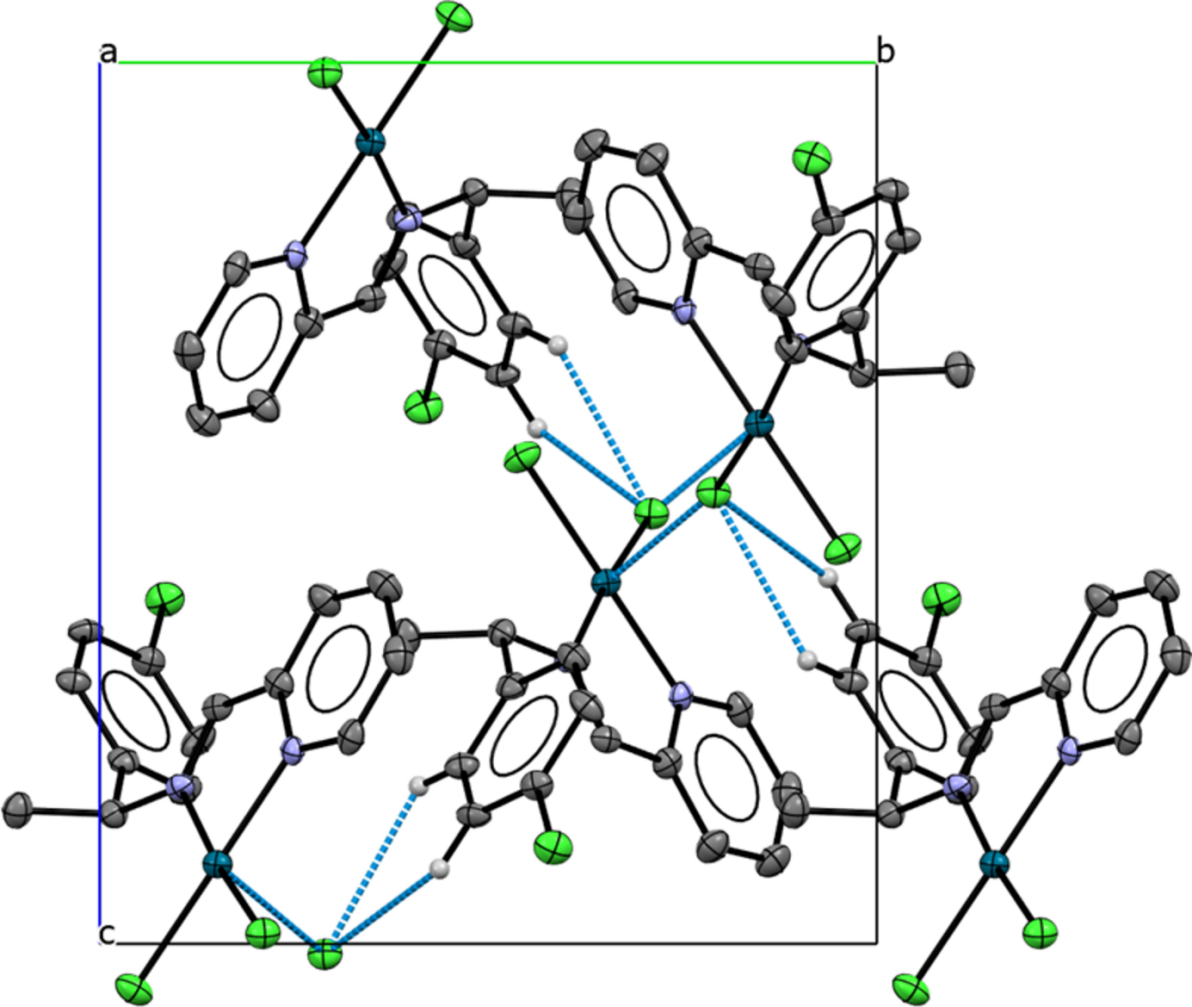
Crystal packing of the Pd^II^ complex; hydrogen bonds are shown as dashed lines. Displacement ellipsoids are drawn at the 30% probability level. All H atoms not involved in these inter­actions have been omitted for clarity

In the crystal of the palladium complex, some short inter­molecular contacts organize the packing into extended motifs. Numerical data of inter­molecular C—H⋯Cl inter­actions are listed in Table 1 ▸ (entries 1–2), and additional inter­actions are observed [Pd1⋯Cl2(*x* − 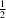, −*y* + 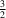, −*z* + 1) = 3.798 (3) Å; Pd1⋯Cl3(−*x* + 2, *y* − 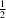, −*z* + 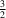) = 3.80 (1) Å]. Weak π–π stacking is present here with centroid-to-centroid separations falling within the typical range of 3.30–3.80 Å: the centroid of the C6–C1–C2–C3–C4–C5 ring is located 3.5915 (5) Å from the centroid of the N2–C14–C13–C12–C11–C10 ring with a slippage of 0.356 Å. A short inter­molecular contact Pd1⋯H13 = 2.905 Å is also identified. All of these contacts contribute to the packing of the crystal (Fig. 4 ▸). The shortest Pd⋯Pd separations exceed 6.00 Å, indicating the absence of significant metal–metal inter­actions.

## 4. Database survey

A search in the Cambridge Structural Database (CSD, Version 5.42, April 2025; Groom *et al.*, 2016 ▸) revealed numerous Pd^II^ complexes featuring N^N bidentate ligands with a square-planar coordination environment. Representative examples include GUTRAS, a palladium complex where the metal is coordinated by pyridine–oxazoline (Pyox) moieties bearing binaftyl and biaryl bridges (Guo *et al.*, 2025 ▸); AJADOH, containing sterically hindered Pyox ligands bound to PdCl_2_ in a *cis* configuration (Chen *et al.*, 2019 ▸); IBEKUY, which displays the typical square-planar environment expected for [Pd^II^Cl_2_*L*] complexes (Gutiérrez *et al.*, 2015 ▸). Complexes ITAJEV, ITAJIZ, and ITAJOF, derived from (imino)­pyridine ligands, show an N^N bidentate coordination mode leading to slightly distorted square-planar environments (Ngcobo *et al.*, 2021 ▸). Similarly, IVIREM exhibits elongated Pd—N bond lengths attributed to steric and electron-donating effects (Tang *et al.*, 2016 ▸). KELRAV contains an *R*-configured Pyox ligand coordinating in a planar fashion (Dodd *et al.*, 2006 ▸). Other examples include MOBSED and MOBSUT, which incorporate hemilabile 2-(1*H*-imidazol-2-yl)pyridine and 2-(oxazol-2-yl)pyridine ligands forming five-membered chelate rings (Eseola *et al.*, 2014 ▸); ONACEO, ONACIS, and ONACOY, which feature moderately bulky Pyox ligands in a square-planar PdCl_2_ array (Tian *et al.*, 2021 ▸); PAGJAJ and PAGJEN show that Pd—N(pyridine) bonds are typically longer than Pd—N(imine) bonds due to the *trans* influence of coordinating substituents (Bastero *et al.*, 2004 ▸). PIKJEA presents a distorted four-coordinate environment involving two chlorido ligands and two nitro­gen donors of an *R*-enanti­omeric ligand (De Crisci *et al.*, 2013 ▸); QASXIR and QASXOX display *cis* chelation and near-ideal square-planar geometry, with short Pd⋯Pd inter­molecular contacts within the crystal structure (Mishnev *et al.*, 2000 ▸); QEJSAZ, where C—H⋯Cl and C—H⋯O inter­actions consolidate the packing of the Pd^II^ complex (Svensson *et al.*, 1999 ▸), RILLUW, corresponding to a PdCl_2_ complex employed in a novel enanti­oselective Pd-catalyzed 6-endo amino­acet­oxy­lation of unactivated alkenes (Qi *et al.*, 2018 ▸); RUCXOF01 and RUCXUL01, involve quinoline-oxazoline (Quox) ligands, which promote a *cis* square-planar coordination environment around Pd^II^. This arrangement has been associated with enhanced enanti­oselective control in catalytic transformations; WUMLEV and WUMLIZ show a slightly tetra­hedrally distorted square-planar environment, with donor atoms deviating from the coordination plane (Bastero *et al.*, 2002 ▸).

## 5. Synthesis and crystallization

Under solvent-free conditions, a 1:1 molar mixture of (*S*)-(−)-1-(4-chloro­phen­yl)ethan-1-amine (0.222 g, 1.42 mmol) and 2-pyridine­carboxaldehyde (0.152 g, 1.42 mmol) was stirred at room temperature, producing a white solid. The crude product was recrystallized twice from hexa­ne/CH_2_Cl_2_ to give colorless crystals of the ligand.

For complex formation, a solution of bis­(benzo­nitrile)­palladium(II) chloride (0.100 g, 0.40 mmol) in CH_2_Cl_2_ (5 ml) was combined with a CH_2_Cl_2_ solution (10 ml) of (*S*)-(−)-*N*-[(2-pyrid­yl)methyl­idene]-1-(4-chloro­phen­yl)ethan-1-amine (0.157 g, 0.40 mmol). The mixture was stirred at room temperature for 24 h, during which a light-orange precipitate formed. The solid was collected by filtration and recrystallized from hexa­ne/CH_2_Cl_2_ to afford light-orange crystals of the Pd^II^ complex.

## 6. Refinement

Crystal data, data collection and structure refinement details are summarized in Table 2 ▸. For H atoms, *U*_iso_(H) was set to 1.2x*U*_eq_ of the parent carbon for CH and aromatic/amide hydrogen atoms, and to 1.5x*U*_eq_ for methyl (CH_3_) groups. Hydrogen atoms were placed in geometrically idealized positions and refined using a riding model: the tertiary CH attached to C7 (H7) was refined as a ternary CH in riding mode, and the aromatic/amide hydrogen atoms attached to C2, C3, C5, C6, C9, C11, C12, C13 and C14 were refined with riding coordinates. The methyl group at C8 (H8*A*, H8*B*, H8*C*) was treated as an idealized methyl and refined as a rotating group.

## Supplementary Material

Crystal structure: contains datablock(s) im-i-l_mo, imilpd_1_mo, New_Global_Publ_Block. DOI: 10.1107/S2056989025011430/wm5775sup1.cif

Structure factors: contains datablock(s) im-i-l_mo. DOI: 10.1107/S2056989025011430/wm5775im-i-l_mosup3.hkl

Structure factors: contains datablock(s) imilpd_1_mo. DOI: 10.1107/S2056989025011430/wm5775imilpd_1_mosup4.hkl

CCDC references: 2517551, 2517552

Additional supporting information:  crystallographic information; 3D view; checkCIF report

## Figures and Tables

**Table 1:** Hydrogen-bond geometry (Å, °) for the PdCl_2_ complex[Chem scheme1]

*D*—H⋯*A*	*D*—H	H⋯*A*	*D*⋯*A*	*D*—H⋯*A*
C2—H2⋯Cl2^i^	0.95	2.83	3.4765 (5)	126
C6—H6⋯Cl2^ii^	0.95	2.73	3.5996 (5)	153
C7—H7⋯Cl1	1.00	2.80	3.318 (10)	113
C8—H8*A*⋯Cl1	0.98	2.78	3.464 (11)	127
C14—H14⋯Cl2	0.95	2.63	3.207 (11)	120

Symmetry codes: (i) 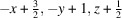; (ii) 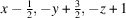.

**Table 2:** Experimental details

	C_14_H_13_ClN_2_	[PdCl_2_(C_14_H_13_ClN_2_)]
Crystal data		
*M*_r_	244.71	422.01
Crystal system, space group	Monoclinic, *P*2_1_	Orthorhombic, *P*2_1_2_1_2_1_
Temperature (K)	293	150
*a*, *b*, *c* (Å)	5.6763 (2), 8.5159 (4), 13.7606 (6)	10.2429 (9), 11.4188 (14), 12.9466 (17)
α, β, γ (°)	90, 89.226 (4), 90	90, 90, 90
*V* (Å^3^)	665.11 (5)	1514.3 (3)
*Z*	2	4
Radiation type	Mo *K*α	Mo *K*α
μ (mm^−1^)	0.27	1.74
Crystal size (mm)	0.7 × 0.59 × 0.08	0.62 × 0.24 × 0.12

Data collection		
Diffractometer	Xcalibur, Atlas, Gemini	Xcalibur, Atlas, Gemini
Absorption correction	Gaussian [*CrysAlis PRO* (Rigaku OD, 2015 ▸) using a multifaceted crystal model based on expressions derived by Clark & Reid (1995 ▸)]	Analytical [*CrysAlis PRO* (Rigaku OD, 2015 ▸) using a multifaceted crystal model based on expressions derived by Clark & Reid (1995 ▸)]
*T*_min_, *T*_max_	0.923, 0.989	0.870, 0.966
No. of measured, independent and observed [*I* > 2σ(*I*)] reflections	41587, 3728, 2109	16724, 3328, 2665
*R*_int_	0.050	0.064
(sin θ/λ)_max_ (Å^−1^)	0.694	0.641

Refinement		
*R*[*F*^2^ > 2σ(*F*^2^)], *wR*(*F*^2^), *S*	0.043, 0.123, 1.01	0.046, 0.092, 1.07
No. of reflections	3728	3328
No. of parameters	155	182
No. of restraints	1	0
H-atom treatment	H-atom parameters constrained	H-atom parameters constrained
Δρ_max_, Δρ_min_ (e Å^−3^)	0.15, −0.19	1.38, −0.67
Absolute structure	Flack *x* determined using 770 quotients [(*I*^+^)−(*I*^−^)]/[(*I*^+^)+(*I*^−^)] (Parsons *et al.*, 2013 ▸)	Flack *x* determined using 1011 quotients [(*I*^+^)−(*I*^−^)]/[(*I*^+^)+(*I*^−^)] (Parsons *et al.*, 2013 ▸)
Absolute structure parameter	−0.03 (3)	−0.06 (3)

Computer programs: *CrysAlis PRO* (Rigaku OD, 2015 ▸), *SHELXS* (Sheldrick, 2008 ▸), *OLEX2.solve* (Bourhis *et al.*, 2015 ▸), *SHELXL* (Sheldrick, 2015 ▸) and *OLEX2* (Dolomanov *et al.*, 2009 ▸).

## supplementary crystallographic information

### (S)-(-)-1-(4-Chlorophenyl)-N-[(pyridin-2-yl)methylidene]ethan-1-amine (im-i-l_mo) Crystal data

**Table:**

C_14_H_13_ClN_2_	*F*(000) = 256
*M**_r_* = 244.71	*D*_x_ = 1.222 Mg m^−^^3^
Monoclinic, *P*2_1_	Mo *K*α radiation, λ = 0.71073 Å
*a* = 5.6763 (2) Å	Cell parameters from 6050 reflections
*b* = 8.5159 (4) Å	θ = 3.6–21.3°
*c* = 13.7606 (6) Å	µ = 0.27 mm^−^^1^
β = 89.226 (4)°	*T* = 293 K
*V* = 665.11 (5) Å^3^	Block, colourless
*Z* = 2	0.7 × 0.59 × 0.08 mm

### (S)-(-)-1-(4-Chlorophenyl)-N-[(pyridin-2-yl)methylidene]ethan-1-amine (im-i-l_mo) Data collection

**Table:**

Xcalibur, Atlas, Gemini diffractometer	2109 reflections with *I* > 2σ(*I*)
Detector resolution: 10.5564 pixels mm^-1^	*R*_int_ = 0.050
ω scans	θ_max_ = 29.6°, θ_min_ = 3.0°
Absorption correction: gaussian [CrysAlisPro (Rigaku OD, 2015) using a multifaceted crystal model based on expressions derived by Clark & Reid (1995)]	*h* = −7→7
*T*_min_ = 0.923, *T*_max_ = 0.989	*k* = −11→11
41587 measured reflections	*l* = −19→19
3728 independent reflections	

### (S)-(-)-1-(4-Chlorophenyl)-N-[(pyridin-2-yl)methylidene]ethan-1-amine (im-i-l_mo) Refinement

**Table:**

Refinement on *F*^2^	Hydrogen site location: inferred from neighbouring sites
Least-squares matrix: full	H-atom parameters constrained
*R*[*F*^2^ > 2σ(*F*^2^)] = 0.043	*w* = 1/[σ^2^(*F*_o_^2^) + (0.0513*P*)^2^ + 0.0411*P*] where *P* = (*F*_o_^2^ + 2*F*_c_^2^)/3
*wR*(*F*^2^) = 0.123	(Δ/σ)_max_ < 0.001
*S* = 1.01	Δρ_max_ = 0.15 e Å^−^^3^
3728 reflections	Δρ_min_ = −0.19 e Å^−^^3^
155 parameters	Absolute structure: Flack *x* determined using 770 quotients [(*I*^+^)-(*I*^-^)]/[(*I*^+^)+(*I*^-^)] (Parsons *et al.*, 2013)
1 restraint	Absolute structure parameter: −0.03 (3)
Primary atom site location: structure-invariant direct methods	

### (S)-(-)-1-(4-Chlorophenyl)-N-[(pyridin-2-yl)methylidene]ethan-1-amine (im-i-l_mo) Special details

**Table:**

Geometry. All esds (except the esd in the dihedral angle between two l.s. planes) are estimated using the full covariance matrix. The cell esds are taken into account individually in the estimation of esds in distances, angles and torsion angles; correlations between esds in cell parameters are only used when they are defined by crystal symmetry. An approximate (isotropic) treatment of cell esds is used for estimating esds involving l.s. planes.

### (S)-(-)-1-(4-Chlorophenyl)-N-[(pyridin-2-yl)methylidene]ethan-1-amine (im-i-l_mo) Fractional atomic coordinates and isotropic or equivalent isotropic displacement parameters (Å^2^)

**Table:**

	*x*	*y*	*z*	*U*_iso_*/*U*_eq_	
C1	0.0976 (5)	0.3440 (3)	0.0974 (2)	0.0655 (7)	
C2	0.0177 (6)	0.4177 (4)	0.0148 (3)	0.0787 (9)	
H2	−0.121009	0.475688	0.018249	0.094*	
C3	0.1379 (6)	0.4075 (4)	−0.0723 (2)	0.0817 (9)	
H3	0.081007	0.457479	−0.127286	0.098*	
C4	0.3412 (5)	0.3233 (4)	−0.0769 (2)	0.0761 (8)	
C5	0.4257 (5)	0.2472 (4)	0.0028 (3)	0.0803 (9)	
H5	0.564010	0.189009	−0.001640	0.096*	
C6	0.3029 (5)	0.2578 (4)	0.0903 (2)	0.0737 (8)	
H6	0.359586	0.206240	0.144757	0.088*	
C7	−0.0352 (6)	0.3630 (4)	0.1930 (2)	0.0787 (9)	
H7	−0.202014	0.381664	0.179750	0.094*	
C8	0.0618 (11)	0.5022 (5)	0.2504 (3)	0.1252 (17)	
H8A	0.222867	0.482028	0.266844	0.188*	
H8B	−0.030054	0.516170	0.308868	0.188*	
H8C	0.053493	0.595577	0.211571	0.188*	
C9	−0.1914 (6)	0.1824 (4)	0.3011 (2)	0.0663 (7)	
H9	−0.329443	0.240387	0.295643	0.080*	
C10	−0.1902 (5)	0.0462 (3)	0.36642 (19)	0.0594 (6)	
C11	−0.0015 (5)	−0.0557 (4)	0.3716 (2)	0.0669 (7)	
H11	0.132864	−0.040192	0.333388	0.080*	
C12	−0.0170 (6)	−0.1810 (4)	0.4350 (2)	0.0814 (9)	
H12	0.106259	−0.252454	0.439271	0.098*	
C13	−0.2142 (6)	−0.1989 (4)	0.4910 (3)	0.0869 (10)	
H13	−0.227604	−0.281927	0.534612	0.104*	
C14	−0.3901 (6)	−0.0935 (5)	0.4818 (3)	0.0886 (11)	
H14	−0.524419	−0.107066	0.520363	0.106*	
Cl1	0.4936 (2)	0.31179 (16)	−0.18694 (8)	0.1191 (5)	
N1	−0.0140 (5)	0.2236 (3)	0.25224 (19)	0.0705 (7)	
N2	−0.3844 (4)	0.0290 (4)	0.42131 (19)	0.0780 (7)	

### (S)-(-)-1-(4-Chlorophenyl)-N-[(pyridin-2-yl)methylidene]ethan-1-amine (im-i-l_mo) Atomic displacement parameters (Å^2^)

**Table:**

	*U*^11^	*U*^22^	*U*^33^	*U*^12^	*U*^13^	*U*^23^
C1	0.0727 (17)	0.0552 (16)	0.0689 (16)	−0.0081 (14)	−0.0095 (13)	0.0102 (13)
C2	0.0786 (19)	0.0687 (19)	0.089 (2)	0.0115 (17)	−0.0045 (17)	0.0194 (17)
C3	0.083 (2)	0.084 (2)	0.078 (2)	0.0062 (18)	−0.0061 (16)	0.0253 (18)
C4	0.0718 (19)	0.079 (2)	0.0778 (19)	−0.0078 (17)	0.0008 (14)	0.0155 (19)
C5	0.0637 (18)	0.085 (2)	0.092 (2)	0.0029 (16)	−0.0035 (16)	0.0198 (19)
C6	0.0710 (18)	0.073 (2)	0.077 (2)	−0.0041 (16)	−0.0151 (15)	0.0194 (16)
C7	0.098 (2)	0.0607 (19)	0.078 (2)	0.0026 (16)	−0.0048 (18)	0.0099 (16)
C8	0.207 (5)	0.075 (3)	0.094 (3)	−0.023 (3)	0.015 (3)	−0.006 (2)
C9	0.0741 (18)	0.0676 (18)	0.0571 (16)	0.0094 (14)	−0.0010 (14)	−0.0044 (14)
C10	0.0662 (16)	0.0598 (15)	0.0521 (14)	−0.0023 (14)	−0.0023 (12)	−0.0068 (13)
C11	0.0653 (16)	0.0674 (18)	0.0679 (18)	0.0005 (15)	0.0007 (13)	−0.0045 (14)
C12	0.086 (2)	0.0700 (19)	0.088 (2)	0.0046 (18)	−0.0147 (17)	0.0074 (19)
C13	0.088 (2)	0.083 (2)	0.090 (2)	−0.013 (2)	−0.0135 (18)	0.026 (2)
C14	0.077 (2)	0.112 (3)	0.077 (2)	−0.013 (2)	0.0066 (16)	0.025 (2)
Cl1	0.1121 (8)	0.1502 (11)	0.0944 (7)	0.0124 (7)	0.0247 (5)	0.0234 (7)
N1	0.0841 (17)	0.0614 (14)	0.0660 (16)	−0.0001 (13)	−0.0015 (13)	0.0072 (12)
N2	0.0670 (15)	0.0952 (19)	0.0717 (16)	0.0036 (14)	0.0064 (12)	0.0052 (16)

### (S)-(-)-1-(4-Chlorophenyl)-N-[(pyridin-2-yl)methylidene]ethan-1-amine (im-i-l_mo) Geometric parameters (Å, º)

**Table:**

C1—C2	1.380 (4)	C8—H8B	0.9600
C1—C6	1.380 (4)	C8—H8C	0.9600
C1—C7	1.516 (4)	C9—H9	0.9300
C2—H2	0.9300	C9—C10	1.467 (4)
C2—C3	1.374 (5)	C9—N1	1.253 (4)
C3—H3	0.9300	C10—C11	1.381 (4)
C3—C4	1.359 (5)	C10—N2	1.336 (3)
C4—C5	1.366 (4)	C11—H11	0.9300
C4—Cl1	1.737 (3)	C11—C12	1.380 (5)
C5—H5	0.9300	C12—H12	0.9300
C5—C6	1.386 (5)	C12—C13	1.359 (4)
C6—H6	0.9300	C13—H13	0.9300
C7—H7	0.9800	C13—C14	1.350 (5)
C7—C8	1.531 (6)	C14—H14	0.9300
C7—N1	1.446 (4)	C14—N2	1.334 (5)
C8—H8A	0.9600		
			
C2—C1—C7	120.1 (3)	C7—C8—H8B	109.5
C6—C1—C2	118.0 (3)	C7—C8—H8C	109.5
C6—C1—C7	121.9 (3)	H8A—C8—H8B	109.5
C1—C2—H2	119.2	H8A—C8—H8C	109.5
C3—C2—C1	121.7 (3)	H8B—C8—H8C	109.5
C3—C2—H2	119.2	C10—C9—H9	118.7
C2—C3—H3	120.5	N1—C9—H9	118.7
C4—C3—C2	119.0 (3)	N1—C9—C10	122.7 (3)
C4—C3—H3	120.5	C11—C10—C9	122.6 (3)
C3—C4—C5	121.3 (3)	N2—C10—C9	115.0 (3)
C3—C4—Cl1	118.9 (3)	N2—C10—C11	122.4 (3)
C5—C4—Cl1	119.8 (3)	C10—C11—H11	120.8
C4—C5—H5	120.4	C12—C11—C10	118.4 (3)
C4—C5—C6	119.3 (3)	C12—C11—H11	120.8
C6—C5—H5	120.4	C11—C12—H12	120.3
C1—C6—C5	120.7 (3)	C13—C12—C11	119.4 (3)
C1—C6—H6	119.6	C13—C12—H12	120.3
C5—C6—H6	119.6	C12—C13—H13	120.8
C1—C7—H7	109.0	C14—C13—C12	118.5 (3)
C1—C7—C8	110.6 (3)	C14—C13—H13	120.8
C8—C7—H7	109.0	C13—C14—H14	117.8
N1—C7—C1	110.9 (3)	N2—C14—C13	124.5 (3)
N1—C7—H7	109.0	N2—C14—H14	117.8
N1—C7—C8	108.2 (3)	C9—N1—C7	117.4 (3)
C7—C8—H8A	109.5	C14—N2—C10	116.9 (3)
			
C1—C2—C3—C4	−0.3 (5)	C8—C7—N1—C9	96.1 (4)
C1—C7—N1—C9	−142.4 (3)	C9—C10—C11—C12	−179.7 (3)
C2—C1—C6—C5	0.6 (5)	C9—C10—N2—C14	−179.9 (3)
C2—C1—C7—C8	−91.5 (4)	C10—C9—N1—C7	−177.9 (3)
C2—C1—C7—N1	148.5 (3)	C10—C11—C12—C13	−1.0 (5)
C2—C3—C4—C5	0.9 (5)	C11—C10—N2—C14	−0.6 (5)
C2—C3—C4—Cl1	−179.6 (3)	C11—C12—C13—C14	0.7 (5)
C3—C4—C5—C6	−0.8 (5)	C12—C13—C14—N2	−0.2 (6)
C4—C5—C6—C1	0.0 (5)	C13—C14—N2—C10	0.2 (5)
C6—C1—C2—C3	−0.4 (5)	Cl1—C4—C5—C6	179.7 (3)
C6—C1—C7—C8	86.6 (4)	N1—C9—C10—C11	−6.7 (4)
C6—C1—C7—N1	−33.4 (4)	N1—C9—C10—N2	172.7 (3)
C7—C1—C2—C3	177.7 (3)	N2—C10—C11—C12	1.0 (4)
C7—C1—C6—C5	−177.5 (3)		

### cis-Dichlorido{(S)-(-)-1-(4-chlorophenyl)-N-[(pyridin-2-yl)methylidene]ethan-1-amine}palladium(II) complex (imilpd_1_mo) Crystal data

**Table:**

[PdCl_2_(C_14_H_13_ClN_2_)]	*D*_x_ = 1.851 Mg m^−^^3^
*M**_r_* = 422.01	Mo *K*α radiation, λ = 0.71073 Å
Orthorhombic, *P*2_1_2_1_2_1_	Cell parameters from 3623 reflections
*a* = 10.2429 (9) Å	θ = 3.6–26.7°
*b* = 11.4188 (14) Å	µ = 1.74 mm^−^^1^
*c* = 12.9466 (17) Å	*T* = 150 K
*V* = 1514.3 (3) Å^3^	Plate, translucent orange
*Z* = 4	0.62 × 0.24 × 0.12 mm
*F*(000) = 832	

### cis-Dichlorido{(S)-(-)-1-(4-chlorophenyl)-N-[(pyridin-2-yl)methylidene]ethan-1-amine}palladium(II) complex (imilpd_1_mo) Data collection

**Table:**

Xcalibur, Atlas, Gemini diffractometer	2665 reflections with *I* > 2σ(*I*)
Detector resolution: 10.5564 pixels mm^-1^	*R*_int_ = 0.064
ω scans	θ_max_ = 27.1°, θ_min_ = 3.1°
Absorption correction: analytical [CrysAlisPro (Rigaku OD, 2015) using a multifaceted crystal model based on expressions derived by Clark & Reid (1995)]	*h* = −13→13
*T*_min_ = 0.870, *T*_max_ = 0.966	*k* = −14→14
16724 measured reflections	*l* = −16→16
3328 independent reflections	

### cis-Dichlorido{(S)-(-)-1-(4-chlorophenyl)-N-[(pyridin-2-yl)methylidene]ethan-1-amine}palladium(II) complex (imilpd_1_mo) Refinement

**Table:**

Refinement on *F*^2^	Hydrogen site location: inferred from neighbouring sites
Least-squares matrix: full	H-atom parameters constrained
*R*[*F*^2^ > 2σ(*F*^2^)] = 0.046	*w* = 1/[σ^2^(*F*_o_^2^) + 8.4403*P*] where *P* = (*F*_o_^2^ + 2*F*_c_^2^)/3
*wR*(*F*^2^) = 0.092	(Δ/σ)_max_ = 0.001
*S* = 1.07	Δρ_max_ = 1.38 e Å^−^^3^
3328 reflections	Δρ_min_ = −0.67 e Å^−^^3^
182 parameters	Absolute structure: Flack *x* determined using 1011 quotients [(*I*^+^)-(*I*^-^)]/[(*I*^+^)+(*I*^-^)] (Parsons *et al.*, 2013)
0 restraints	Absolute structure parameter: −0.06 (3)
Primary atom site location: iterative	

### cis-Dichlorido{(S)-(-)-1-(4-chlorophenyl)-N-[(pyridin-2-yl)methylidene]ethan-1-amine}palladium(II) complex (imilpd_1_mo) Special details

**Table:**

Geometry. All esds (except the esd in the dihedral angle between two l.s. planes) are estimated using the full covariance matrix. The cell esds are taken into account individually in the estimation of esds in distances, angles and torsion angles; correlations between esds in cell parameters are only used when they are defined by crystal symmetry. An approximate (isotropic) treatment of cell esds is used for estimating esds involving l.s. planes.

### cis-Dichlorido{(S)-(-)-1-(4-chlorophenyl)-N-[(pyridin-2-yl)methylidene]ethan-1-amine}palladium(II) complex (imilpd_1_mo) Fractional atomic coordinates and isotropic or equivalent isotropic displacement parameters (Å^2^)

**Table:**

	*x*	*y*	*z*	*U*_iso_*/*U*_eq_	
C1	0.4453 (10)	0.5307 (9)	0.7078 (7)	0.033 (2)	
C2	0.4209 (10)	0.4661 (9)	0.7983 (7)	0.033 (2)	
H2	0.483692	0.410719	0.821343	0.040*	
C3	0.3073 (10)	0.4815 (9)	0.8542 (7)	0.033 (2)	
H3	0.291881	0.437210	0.915123	0.039*	
C4	0.2163 (9)	0.5625 (9)	0.8203 (8)	0.032 (2)	
C5	0.2377 (9)	0.6267 (9)	0.7300 (8)	0.038 (3)	
H5	0.174424	0.681322	0.706440	0.046*	
C6	0.3530 (10)	0.6095 (9)	0.6748 (8)	0.037 (3)	
H6	0.367948	0.653067	0.613396	0.044*	
C7	0.5713 (10)	0.5176 (8)	0.6472 (8)	0.031 (2)	
H7	0.549819	0.533739	0.573198	0.038*	
C8	0.6299 (10)	0.3943 (9)	0.6513 (9)	0.039 (3)	
H8A	0.706730	0.390586	0.606362	0.059*	
H8B	0.564812	0.337268	0.627834	0.059*	
H8C	0.655573	0.376058	0.722453	0.059*	
C9	0.6734 (9)	0.6515 (8)	0.7680 (6)	0.0279 (17)	
H9	0.605894	0.634992	0.816155	0.033*	
C10	0.7778 (9)	0.7307 (9)	0.7950 (8)	0.031 (2)	
C11	0.7857 (10)	0.7891 (9)	0.8896 (8)	0.040 (3)	
H11	0.722366	0.775686	0.941951	0.049*	
C12	0.8873 (9)	0.8666 (9)	0.9058 (10)	0.044 (3)	
H12	0.895917	0.906679	0.969730	0.053*	
C13	0.9756 (11)	0.8846 (9)	0.8274 (9)	0.042 (3)	
H13	1.044741	0.939019	0.836551	0.050*	
C14	0.9644 (9)	0.8235 (8)	0.7345 (8)	0.034 (2)	
H14	1.027010	0.836022	0.681529	0.041*	
Cl1	0.7729 (3)	0.5439 (3)	0.4479 (2)	0.0410 (6)	
Cl2	1.0184 (2)	0.7101 (2)	0.51194 (19)	0.0354 (6)	
Cl3	0.0736 (2)	0.5835 (2)	0.8907 (2)	0.0430 (7)	
N1	0.6727 (8)	0.6036 (6)	0.6781 (5)	0.0281 (16)	
N2	0.8678 (7)	0.7481 (6)	0.7191 (6)	0.0265 (17)	
Pd1	0.82980 (7)	0.65153 (6)	0.59030 (5)	0.02722 (17)	

### cis-Dichlorido{(S)-(-)-1-(4-chlorophenyl)-N-[(pyridin-2-yl)methylidene]ethan-1-amine}palladium(II) complex (imilpd_1_mo) Atomic displacement parameters (Å^2^)

**Table:**

	*U*^11^	*U*^22^	*U*^33^	*U*^12^	*U*^13^	*U*^23^
C1	0.034 (6)	0.038 (6)	0.028 (5)	−0.004 (4)	−0.008 (4)	−0.004 (4)
C2	0.027 (5)	0.044 (6)	0.028 (5)	−0.002 (5)	−0.003 (4)	−0.003 (5)
C3	0.030 (6)	0.045 (6)	0.023 (5)	−0.010 (5)	0.002 (4)	0.002 (4)
C4	0.024 (5)	0.039 (6)	0.033 (5)	−0.004 (4)	0.003 (4)	−0.001 (5)
C5	0.028 (5)	0.046 (7)	0.042 (6)	0.000 (4)	−0.004 (4)	0.016 (5)
C6	0.034 (6)	0.040 (6)	0.036 (5)	−0.005 (5)	−0.003 (5)	0.007 (4)
C7	0.031 (5)	0.032 (6)	0.031 (5)	−0.005 (4)	−0.003 (4)	0.000 (4)
C8	0.035 (6)	0.035 (6)	0.048 (7)	−0.003 (5)	0.006 (5)	0.003 (5)
C9	0.021 (4)	0.035 (5)	0.027 (4)	0.008 (5)	0.000 (4)	0.002 (4)
C10	0.025 (5)	0.034 (6)	0.036 (6)	0.007 (4)	0.000 (4)	−0.002 (5)
C11	0.037 (6)	0.043 (6)	0.042 (7)	0.002 (5)	0.006 (5)	−0.009 (5)
C12	0.044 (6)	0.044 (6)	0.043 (6)	0.006 (5)	0.003 (6)	−0.013 (7)
C13	0.033 (6)	0.035 (6)	0.058 (7)	−0.003 (5)	−0.009 (5)	−0.003 (5)
C14	0.026 (5)	0.029 (6)	0.047 (6)	0.008 (4)	0.005 (4)	0.006 (5)
Cl1	0.0353 (13)	0.0519 (17)	0.0358 (13)	−0.0044 (12)	0.0051 (11)	−0.0131 (12)
Cl2	0.0282 (13)	0.0432 (15)	0.0350 (13)	−0.0025 (11)	0.0019 (10)	0.0007 (11)
Cl3	0.0304 (13)	0.0540 (17)	0.0447 (17)	0.0026 (12)	0.0069 (12)	0.0025 (13)
N1	0.027 (4)	0.032 (4)	0.025 (4)	0.009 (4)	0.006 (4)	0.002 (3)
N2	0.028 (4)	0.021 (4)	0.031 (4)	0.007 (3)	0.000 (3)	−0.001 (3)
Pd1	0.0225 (3)	0.0317 (3)	0.0274 (3)	0.0018 (3)	0.0002 (3)	−0.0006 (3)

### cis-Dichlorido{(S)-(-)-1-(4-chlorophenyl)-N-[(pyridin-2-yl)methylidene]ethan-1-amine}palladium(II) complex (imilpd_1_mo) Geometric parameters (Å, º)

**Table:**

C1—C2	1.407 (14)	C9—H9	0.9500
C1—C6	1.374 (14)	C9—C10	1.443 (13)
C1—C7	1.518 (14)	C9—N1	1.287 (10)
C2—H2	0.9500	C10—C11	1.397 (13)
C2—C3	1.382 (13)	C10—N2	1.362 (12)
C3—H3	0.9500	C11—H11	0.9500
C3—C4	1.385 (13)	C11—C12	1.382 (14)
C4—C5	1.398 (13)	C12—H12	0.9500
C4—Cl3	1.739 (10)	C12—C13	1.375 (15)
C5—H5	0.9500	C13—H13	0.9500
C5—C6	1.394 (14)	C13—C14	1.395 (14)
C6—H6	0.9500	C14—H14	0.9500
C7—H7	1.0000	C14—N2	1.327 (12)
C7—C8	1.531 (13)	Cl1—Pd1	2.291 (3)
C7—N1	1.484 (12)	Cl2—Pd1	2.282 (3)
C8—H8A	0.9800	N1—Pd1	2.045 (8)
C8—H8B	0.9800	N2—Pd1	2.036 (8)
C8—H8C	0.9800		
			
C2—C1—C7	122.0 (9)	C10—C9—H9	120.3
C6—C1—C2	118.7 (10)	N1—C9—H9	120.3
C6—C1—C7	119.3 (9)	N1—C9—C10	119.3 (9)
C1—C2—H2	119.4	C11—C10—C9	123.6 (9)
C3—C2—C1	121.3 (10)	N2—C10—C9	114.7 (8)
C3—C2—H2	119.4	N2—C10—C11	121.6 (9)
C2—C3—H3	120.5	C10—C11—H11	120.6
C2—C3—C4	119.1 (9)	C12—C11—C10	118.8 (10)
C4—C3—H3	120.5	C12—C11—H11	120.6
C3—C4—C5	120.6 (9)	C11—C12—H12	120.7
C3—C4—Cl3	119.5 (8)	C13—C12—C11	118.6 (11)
C5—C4—Cl3	119.9 (8)	C13—C12—H12	120.7
C4—C5—H5	120.4	C12—C13—H13	119.7
C6—C5—C4	119.2 (9)	C12—C13—C14	120.5 (10)
C6—C5—H5	120.4	C14—C13—H13	119.7
C1—C6—C5	121.1 (9)	C13—C14—H14	119.5
C1—C6—H6	119.5	N2—C14—C13	121.0 (9)
C5—C6—H6	119.5	N2—C14—H14	119.5
C1—C7—H7	106.8	C7—N1—Pd1	125.3 (6)
C1—C7—C8	114.0 (8)	C9—N1—C7	122.0 (8)
C8—C7—H7	106.8	C9—N1—Pd1	112.6 (7)
N1—C7—C1	112.9 (8)	C10—N2—Pd1	112.5 (6)
N1—C7—H7	106.8	C14—N2—C10	119.4 (8)
N1—C7—C8	109.0 (8)	C14—N2—Pd1	128.1 (7)
C7—C8—H8A	109.5	Cl2—Pd1—Cl1	90.85 (9)
C7—C8—H8B	109.5	N1—Pd1—Cl1	95.9 (2)
C7—C8—H8C	109.5	N1—Pd1—Cl2	172.6 (2)
H8A—C8—H8B	109.5	N2—Pd1—Cl1	176.3 (2)
H8A—C8—H8C	109.5	N2—Pd1—Cl2	92.5 (2)
H8B—C8—H8C	109.5	N2—Pd1—N1	80.8 (3)
			
C1—C2—C3—C4	−0.1 (15)	C9—C10—C11—C12	−177.5 (9)
C1—C7—N1—C9	24.3 (12)	C9—C10—N2—C14	177.0 (8)
C1—C7—N1—Pd1	−159.7 (6)	C9—C10—N2—Pd1	−1.8 (10)
C2—C1—C6—C5	0.7 (15)	C10—C9—N1—C7	177.4 (8)
C2—C1—C7—C8	32.0 (13)	C10—C9—N1—Pd1	0.9 (10)
C2—C1—C7—N1	−93.0 (11)	C10—C11—C12—C13	0.9 (15)
C2—C3—C4—C5	0.9 (15)	C11—C10—N2—C14	−1.0 (13)
C2—C3—C4—Cl3	−179.5 (7)	C11—C10—N2—Pd1	−179.8 (7)
C3—C4—C5—C6	−0.9 (15)	C11—C12—C13—C14	−1.6 (16)
C4—C5—C6—C1	0.1 (16)	C12—C13—C14—N2	0.9 (15)
C6—C1—C2—C3	−0.7 (15)	C13—C14—N2—C10	0.4 (13)
C6—C1—C7—C8	−149.2 (10)	C13—C14—N2—Pd1	178.9 (7)
C6—C1—C7—N1	85.8 (11)	Cl3—C4—C5—C6	179.5 (8)
C7—C1—C2—C3	178.1 (9)	N1—C9—C10—C11	178.5 (9)
C7—C1—C6—C5	−178.1 (9)	N1—C9—C10—N2	0.6 (13)
C8—C7—N1—C9	−103.4 (10)	N2—C10—C11—C12	0.3 (15)
C8—C7—N1—Pd1	72.6 (9)		

### cis-Dichlorido{(S)-(-)-1-(4-chlorophenyl)-N-[(pyridin-2-yl)methylidene]ethan-1-amine}palladium(II) complex (imilpd_1_mo) Hydrogen-bond geometry (Å, º)

**Table:**

*D*—H···*A*	*D*—H	H···*A*	*D*···*A*	*D*—H···*A*
C2—H2···Cl2^i^	0.95	2.83	3.4765 (5)	126
C6—H6···Cl2^ii^	0.95	2.73	3.5996 (5)	153
C7—H7···Cl1	1.00	2.80	3.318 (10)	113
C8—H8*A*···Cl1	0.98	2.78	3.464 (11)	127
C14—H14···Cl2	0.95	2.63	3.207 (11)	120

Symmetry codes: (i) −*x*+3/2, −*y*+1, *z*+1/2; (ii) *x*−1/2, −*y*+3/2, −*z*+1.

## References

[bb1] Anzaldo Olivares, B., Moreno, O. P., Téllez, G. H., Rosas, E. R., Bustamante, F. J. M., Castro Sánchez, M. E., Sharma, P., Mendoza, A. & Pérez, R. G. (2019). *Opt. Mater.* **94**, 337–347.

[bb2] Bastero, A., Claver, C., Ruiz, A., Castillón, S., Daura, E., Bo, C. & Zangrando, E. (2004). *Chem. A Eur. J.* **10**, 3747–3760. 10.1002/chem.200306051 15281159

[bb3] Bastero, A., Ruiz, A., Claver, C., Milani, B. & Zangrando, E. (2002). *Organometallics* **21**, 5820–5829.

[bb4] Bourhis, L. J., Dolomanov, O. V., Gildea, R. J., Howard, J. A. K. & Puschmann, H. (2015). *Acta Cryst.* A**71**, 59–75. 10.1107/S2053273314022207 PMC4283469 25537389

[bb5] Bugarčić, Ž. D., Bogojeski, J. & van Eldik, R. (2015). *Coord. Chem. Rev.* **292**, 91–106.

[bb6] Chen, C., Pflüger, P. M., Chen, P. & Liu, G. (2019). *Angew. Chem. Int. Ed.* **58**, 2392–2396. 10.1002/anie.201813591 30694601

[bb7] Clark, R. C. & Reid, J. S. (1995). *Acta Cryst.* A**51**, 887–897.

[bb8] Dalia, S. A., Afsan, F., Hossain, M. S., Khan, M. N., Zakaria, C., Zahan, M. E. & Ali, M. (2018). *J. Chem. Stud.* **6**, 2859–2867.

[bb9] De Crisci, A. G., Chung, K., Oliver, A. G., Solis-Ibarra, D. & Waymouth, R. M. (2013). *Organometallics* **32**, 2257–2266.

[bb10] Dodd, D. W., Toews, H. E., Carneiro, F., d, S., Jennings, M. C. & Jones, N. D. (2006). *Inorg. Chim. Acta* **359**, 2850–2858.

[bb11] Dolomanov, O. V., Bourhis, L. J., Gildea, R. J., Howard, J. A. K. & Puschmann, H. (2009). *J. Appl. Cryst.* **42**, 339–341.

[bb12] Eseola, A. O., Geibig, D., Görls, H., Sun, W.-H., Hao, X., Woods, J. A. O. & Plass, W. (2014). *J. Organomet. Chem.* **754**, 39–50.

[bb13] Fabbrizzi, L. (2020). *J. Org. Chem.* **85**, 12212–12226. 10.1021/acs.joc.0c01420 PMC8011914 32864964

[bb14] Groom, C. R., Bruno, I. J., Lightfoot, M. P. & Ward, S. C. (2016). *Acta Cryst.* B**72**, 171–179. 10.1107/S2052520616003954 PMC4822653 27048719

[bb15] Guo, B., Yan, X., Wang, Z., Shen, C., Chen, W., Cen, S., Peng, Q. & Zhang, Z. (2025). *J. Am. Chem. Soc.* **147**, 12614–12626. 10.1021/jacs.5c00077 40167529

[bb16] Gutiérrez, D., Bernès, S., Hernández, G., Portillo, O., Moreno, G. E., Sharma, M., Sharma, P. & Gutiérrez, R. (2015). *J. Coord. Chem.* **68**, 3805–3813.

[bb17] Hernández Téllez, G., Reyes-Avendaño, J. A., Bravo-Arredondo, J. M., Moreno Morales, G. E., Sharma, P., Villamizar, C. P., Mendoza, A. & Anzaldo, B. (2025). *ACS Omega* **10**, 51170–51185. 10.1021/acsomega.5c06111 PMC12593969 41210815

[bb18] Mandal, P. & Pratihar, J. L. (2023). *Rev. Inorg. Chem.* **43**, 415–436.

[bb19] Mishnev, A., Iovel, I., Popelis, J., Vosekalna, I. & Lukevics, E. (2000). *J. Organomet. Chem.* **608**, 1–5.

[bb20] Ngcobo, N. L., Akiri, S. O., Ogweno, A. O. & Ojwach, S. O. (2021). *Polyhedron* **203**, 115243–115243.

[bb21] Parsons, S., Flack, H. D. & Wagner, T. (2013). *Acta Cryst.* B**69**, 249–259. 10.1107/S2052519213010014 PMC3661305 23719469

[bb22] Qi, X., Chen, C., Hou, C., Fu, L., Chen, P. & Liu, G. (2018). *J. Am. Chem. Soc.* **140**, 7415–7419. 10.1021/jacs.8b03767 29812946

[bb23] Rigaku OD (2015). *CrysAlis PRO*. Rigaku Oxford Diffraction Ltd, Yarnton, England.

[bb24] Sheldrick, G. M. (2008). *Acta Cryst.* A**64**, 112–122. 10.1107/S0108767307043930 18156677

[bb25] Sheldrick, G. M. (2015). *Acta Cryst.* C**71**, 3–8.

[bb26] Svensson, M., Bremberg, U., Hallman, K., Csöregh, I. & Moberg, C. (1999). *Organometallics* **18**, 4900–4907.

[bb27] Takeda, C., Nakane, D. & Akitsu, T. (2023). *Molecules* **28**, 7990–7990. 10.3390/molecules28247990 PMC10745973 38138480

[bb28] Tang, Y., Zeng, Y., Hu, Q., Huang, F., Jin, L., Mo, W., Sun, N., Hu, B., Shen, Z., Hu, X. & Sun, W.-H. (2016). *Adv. Synth. Catal.* **358**, 2642–2651.

[bb29] Tian, B., Chen, P., Leng, X. & Liu, G. (2021). *Nat. Catal.* **4**, 172–179.

